# Relationship between chewing tobacco, smoking, consuming alcohol and cognitive impairment among older adults in India: a cross‐sectional study

**DOI:** 10.1186/s12877-021-02027-x

**Published:** 2021-01-29

**Authors:** T. Muhammad, Manideep Govindu, Shobhit Srivastava

**Affiliations:** International Institute for Population Sciences, 400088 Mumbai, Maharashtra India

## Abstract

**Background:**

Physical aging increases the sensitivity to the effects of substance use, elevating the risk for cognitive impairment among older adults. Since studies on the association of substance use with cognitive ability in later years are scant in India, we aimed to explore the factors associated with cognitive impairment especially, alcohol consumption, smoking, and chewing tobacco later in life.

**Methods:**

The present research used nationally representative data from Building a Knowledge Base on Population Aging in India (BKPAI) that was conducted in 2011, across seven states of India (N=9,453). Sample distribution along with percentage distribution was calculated for cognitive impairment over explanatory variables. For finding the association between cognitive impairment over explanatory variables, binary logistic regression models were estimated.

**Results:**

About 16.5 percent of older adults in rural areas consumed smoked tobacco compared to 11.7 percent in urban areas. Nearly, 23.7 percent of rural older adults consumed smokeless tobacco in comparison to 16 percent in urban areas. Alcohol consumption was high among rural residents (7.9%) than urban counterparts (6.7%). The prevalence of cognitive impairment was 62.8% and 58% among older adults from rural and urban areas respectively. Older adults who smoked tobacco had a 24 percent significantly higher likelihood to have cognitive impairment with reference to older adults who did not smoke [OR: 1.24, CI: 1.02-1.49]. Moreover, older adults who consumed alcohol had a 30 percent significantly higher likelihood to have cognitive impairment [OR: 1.02, 1.65]. It was also found that older adults who had smoked along with consuming alcohol were at risk of worse cognitive outcomes than those who neither smoke nor drink alcohol [OR: 1.56, CI: 1.21-2.00] or consumed either of them unlike consuming smokeless tobacco only.

**Conclusion:**

The encouragement of older people to stop smoking and smokeless tobacco use could be considered as part of a strategy to reduce the incidence of cognitive impairment. Further, appropriate measures should be taken for the detection of early stages of cognitive decline in older individuals and efforts should be made to improve the availability and quality of care for dementing older adults.

## Background

The longer people live, the greater the risk of aging biology in determining both length and quality of life [1]. Older adults are placed at increased risk of substance use disorders due to environmental and social factors associated with aging [2, 3]. Similarly, physical aging and commonly used medications can result in increased sensitivity to the effects of substance use, elevating the risk for cognitive impairment [4–7].

In 2016, Alzheimer’s disease and other dementias appeared for the first time as ranked fifth non-communicable disease in the World Health Organization (WHO) top 10 causes of death globally [8]. A systematic review revealed that the proportion of dementia that is characterized as the severe decline in cognitive functioning and the prevalence of cognitive impairment is increasing in developing countries [9]. Moreover, the study observed that although it is difficult to separate the effects of normal aging from that of disease, it appears that intellectual decline in some cognitive domains is an inevitable consequence of aging [10]. Further, a dearth of literature showed that the link between substance use disorders and objective cognitive outcomes has been well covered [11–13]. However, whether or not alcohol use is associated with cognition among older individuals is an unresolved issue.

### Alcohol drinking and tobacco use in later life

The study found that heavy alcohol consumption in older adults has been associated with a faster decline in cognition in late middle age, particularly in men [14]. At the same time, evidence shows that consuming alcohol has a positive effect on several health outcomes. Interestingly, studies from developed as well as developing countries have shown that consuming alcohol at a mild to moderate level acts as a protective factor against cognitive decline among older adults, compared to excessive consumption of alcohol [15–17]. Although a number of studies have shown inconsistent results, light to moderate alcohol use among older people in some studies appeared to reduce the risk of dementia and Alzheimer’s disease [18–20]. However, researchers have found that alcohol had a negative cognitive impact, even at moderate levels [21]. Many authors have argued about the causal role of substance use in exacerbating psychotic reactions [22]. Further, the study also showed that in psychotic patients, alcohol drinking and smoking may aggravate thought disturbances [23–25]. Similarly, as a result of increased alcohol dependence, the family life is harmed and had a strained relationship with other family members as well as with their neighbour’s due to inappropriate behavior [26, 27] that may have led to poor health outcomes including cognition. Besides, a history of alcohol dependence, even when it is not associated with current heavy alcohol use, can also be associated with persistent cognitive impairment [28].

Furthermore, a recent study found that between 2010 and 2017, alcohol consumption in India increased by 38 percent from 4.3 to 5.9 liters per adult per year [29]. On the other hand, according to Global Adults Tobacco Survey India (GATS India, 2016–2017), in India, 41.4 percent of adults aged 65 years and older currently use tobacco (smoked and/or smokeless tobacco) [30]. The study also observed that tobacco use has been socially accepted among adults and older adults in most Indian societies and is a source of social interaction and recreational pursuit [31]. Unlike western countries, India has a higher number of smokeless tobacco consumers in comparison to smokers. Studies in India showed that smoking and chewing tobacco significantly correlated with the prevalence of coronary heart disease and hypertension and higher quality of life could be achieved by avoiding such habits [32, 33]. It is also demonstrated that tobacco use arguably accounts for far more medical disability and mortality in the older population than abuse of all other substances combined [2]. These findings serve to determine the effects of regular smoking on adverse health outcomes. Although daily smoking seemed to be associated with increased failures [34], it is difficult to disentangle the effects of cumulative use of tobacco over a lifetime on cognitive ability among older adults.

There is also great variation in levels of alcohol consumption, smoking and chewing tobacco and substance disorders across different demographic and socioeconomic groups in India. For instance, a comparative study of Bangladesh, India, and Nepal revealed that the prevalence of smokeless tobacco is higher among adult men in India than among women [35]. Previous work on mental, neurological and substance use disorders has posited that low education and poverty were associated with a higher occurrence of dementia in both China and India [36]. Again, people from lowest levels of socioeconomic groups were prone to report higher rates of smoking than their counterparts [37–39]. Study also suggests that populations with the poorest income status who increasingly purchase smokeless tobacco make use of scarce resources available with them and may have important indirect effects on their overall health [40]. Additionally, as a result of huge socioeconomic variations in the population, a recent study in India also found that several socio-economic and health factors such as increasing age, no schooling, and bedridden status for the past six months were significantly associated with higher cognitive impairment among the older population [41].

However, studies on the association of substance use with cognitive impairment among older adults are scant, especially in a country with low educational attainment. Thus, we aimed to explore the factors associated with the decline in cognitive functioning especially, alcohol consumption, smoking, and chewing tobacco. The study also sought to determine whether smoking/chewing tobacco and alcohol consumption together are associated with greater impairments in cognitive functioning in old age. The study hypothesizes that:

1. Chewing tobacco, alcohol consumption, and smoking are positively associated with cognitive impairment in an aging population.
2. There is a significant interaction between smoked/smokeless tobacco use and alcohol consumption on cognitive impairment among older adults.

## Methods

### Data

The present research used data from Building a Knowledge Base on Population Aging in India (BKPAI) which was a nationally representative data was conducted in 2011, across seven states of India [42]. The survey was sponsored by the Tata Institute for Social Sciences (TISS), Mumbai, and Institute for Economic Growth (IEG), Delhi, Institute for Social and Economic Change (ISEC), and UNFPA (United Nations Population Fund), New Delhi. The survey gathered information on various socio-demographic, economic and health aspects of aging among households of those aged 60 years and above. The data from all the seven states were collected which represents the various regions of India. The states of Punjab and Himachal represent the northern part, Kerala and Tamil Nadu represent the southern part, Orissa and West Bengal represent the eastern part, and Maharashtra represents the western part of the country.

Being the survey of the older adults, the sample size was equally split between urban and rural areas, irrespective of the proportion of the urban and rural population. Eighty Primary Sampling Units (PSU) (villages or urban wards) – 40 urban and an equal number of rural – with 16 households per Primary Sampling Unit (PSU) having an older person were covered in the survey [42]. In all, 9850 older adults were interviewed from 8329 households aged 60 years and above. The sample included for the analysis after dropping the missing data (397 older adults) and outliers was 9453 older adults.

### Variable description

#### Outcome variable

Cognitive impairment was measured by the number of words recalled. To measure the cognitive impairment, a scale of 0 to 10 was prepared. Higher score represents lower cognitive impairment and vice-versa. The words used for testing cognitive impairment were Bus, House, Chair, Banana, Sun, Bird, Cat, Saree, Rice, and Monkey. Five or more words were recoded as 0 “low” representing lower cognitive impairment and a score of four or less was recoded as 1 “high” representing higher cognitive impairment [43–45]. High cognitive impairment represents cognitive disability among older adults in the present study. Place of Residence was recoded as rural and urban. The study was stratified into rural and urban place of residence. However, during multivariate analysis place of residence was used as a control variable to see the adjusted effects.

#### Explanatory variable

There were three main explanatory variables for the study. 1. Smoking tobacco was recoded as 0 “no” and 1 “yes”. 2. Chewing tobacco was recoded as 0 “no” and 1 “yes”. 3. Alcohol consumption was recoded as 0 “no” and 1 “yes”. The questions assessed ‘ever use of smoking tobacco’, ‘chewing tobacco’ and ‘alcohol consumption’.

Age was recoded as 60–69, 70–79, and 80 + years [46]. Sex was recoded as Men and Women. Educational status was recoded as no schooling, below five years of schooling, 6–10 years of schooling, and 11 and above years of schooling. Working status was recoded as “yes”, “no” and “retired”. Marital status was recoded as currently in union and not in union “included never married, widowed, divorced and separated” [47]. Five questions for community involvement were asked and were used to create a variable to measure social capital. The score developed ranges from 0 to 5 and a score of 1 to 5 was recoded as 0 “community involvement” a score of 0 was recoded as 1 “no community involvement”. The other question “do you have someone you can trust and confide in?” was recoded into binary form as 0 “yes” and 1 “no” [47]. Living arrangement was recoded as “others”, “living alone and with spouse” [47]. Self-rated health had a scale of 1 to 5 “poor to excellent” and was recoded as 0 “good” (representing good, very good, and excellent) and 1 “poor” (representing poor or fair) [44, 47]. Chronic morbidity was recoded as 0 “no” and 1 “yes”. Ability to do activities of daily living had a scale of 0 to 6 where it represents higher the score, higher the independence. A score of was recoded as 0 “high” which represents complete independence and 5 and less was recoded as 1 “low” which represents not completely independent to do activities of daily living (Cronbach Alpha: 0.93) [48]. Ability to do instrumental activities of daily living had a scale of 0 to 8 representing higher the score, higher the independence. A score of 6 + was recoded as 0 “high” representing high IADL and a score of 5 and less was recoded as 1 “low” representing low IADL [42, 44, 46]. The International Classification of Functioning, Disability, and Health (ICF) proposed the framework on which ADL and IADL were calculated. The Activities of Daily Living (ADL) is an umbrella term relating to self-care, comprising those activities that people undertake routinely in their everyday life. The activities can be subdivided into personal care or ADL and domestic and community activities or Instrumental ADL (IADL). The ADL and IADL have emerged as the most common approaches in empirical assessments of functionality among the older adults and are considered to be befitting to the ICF framework [49].

Caste was recoded as non-Scheduled Caste/Scheduled Tribes and Scheduled Caste/Scheduled Tribes (SC/ST) [50]. Religion was recoded as Hindu, Muslim, and others. Wealth status was computed using 30 household assets and was divided into 5 quintiles as poorest, poorer, middle, richer, richest. Then the wealth index was divided as poor “poorest/poorer”, middle and rich “richer/richest” [42]. Data for seven states was available in the data as mentioned in the data section.

### Statistical analysis

Using STATA 14 [51] sample distribution along with percentage distribution was calculated for cognitive impairment over explanatory variables. For finding the association between cognitive impairment over explanatory variables binary logistic regression model [52] was used. The outcome variable was cognitive impairment coded as “low (0) and high (1)” and the main explanatory variables were consumption of tobacco (smoking and chewing) and consumption of alcohol.

The binary logistic regression model is usually put into a more compact form as follows:

$$ \mathrm{Logit}\left[\mathrm{P}\left(\mathrm{Y}=1\right)\right]={\beta}_0+\beta \ast X+\in $$

The parameter *β*_0_ estimates the log odds of the cognitive impairment for the reference group, while *β* estimates the maximum likelihood, the differential log odds of the cognitive impairment associated with set of predictors X, as compared to the reference group and ϵ represents the residual in the model.

The multivariate analysis had four models to explain the unadjusted and adjusted estimates. Model-1 was used to provide the independent effect of smoking tobacco, chewing tobacco, and alcohol consumption on cognitive impairment of older adults. Model-2 was an adjusted model (full-effect model) providing adjusted estimates. The model was adjusted for socio-economic and background characteristics. Model-3, Model-4, and Model-5 provide interaction effects [45, 53, 54] for those who smoke tobacco and consume alcohol; chew tobacco and consume alcohol and who smoke and chew tobacco on cognitive impairment among older adults.

## Results

Table 1 represents the socio-economic profile of older adults in India. About 16.5 percent of older adults consumed smoked tobacco in reference to 11.7 percent in urban areas. Nearly, 23.7 percent of older adults consumed chewing tobacco in comparison to 16 percent in urban areas.

**Table 1:** Socio-economic profile of older adults in India

**Variables**	**Rural**		**Urban**	
	**Sample**	**Percentage**	**Sample**	**Percentage**
**Smoking tobacco**				
No	4128	83.5	3980	88.3
Yes	816	16.5	529	11.7
**Chewing tobacco**				
No	3772	76.3	3788	84.0
Yes	1172	23.7	721	16.0
**Alcohol consumption**				
No	4552	92.1	4207	93.3
Yes	392	7.9	302	6.7
**Age (years)**				
60–69	2954	59.7	3035	67.3
70–79	1429	28.9	1053	23.4
80+	562	11.4	421	9.3
**Sex**				
Men	2366	47.9	2077	46.1
Women	2578	52.1	2432	53.9
**Education**				
No education	2858	57.8	1443	32.0
Below 5 years	1026	20.8	892	19.8
6–10 years	859	17.4	1643	36.4
11 + years	201	4.1	531	11.8
**Working status**				
Yes	1299	26.3	835	18.5
No	3342	67.6	2983	66.2
Retired	303	6.1	691	15.3
**Marital Status**				
Currently in Union	3060	61.9	2561	56.8
Not in Union	1884	38.1	1948	43.2
**Community involvement**				
Yes	3857	78.0	3710	82.3
No	1087	22.0	799	17.7
**Trust over someone**				
Yes	4023	81.4	3922	87.0
No	921	18.6	587	13.0
**Living arrangement**				
Others	3799	76.9	3631	80.5
Living alone/with spouse	1145	23.2	878	19.5
**Self-rated health**				
Good	2138	43.3	2188	48.5
Poor	2806	56.8	2321	51.5
**Chronic morbidity**				
No	1700	32.4	1691	37.5
Yes	3244	65.6	2819	62.5
**ADL**				
High	4565	92.3	4209	93.4
Low	379	7.7	300	6.6
**IADL**				
High	2002	40.5	2318	51.4
Low	2942	59.5	2191	48.6
**Caste**				
Non-SC/ST	3493	70.7	3700	82.1
SC/ST	1451	29.3	809	18.0
**Religion**				
Hindu	3954	80.0	3535	78.4
Muslim	307	6.2	415	9.2
Others	683	13.8	560	12.4
**Wealth index**				
Rich	1300	26.3	2401	53.3
Middle	962	19.5	1086	24.1
Poor	2681	54.2	1020	22.6
**States**				
Himachal Pradesh	978	19.8	133	3.0
Punjab	672	13.6	657	14.6
West Bengal	528	10.7	692	15.4
Orissa	908	18.4	308	6.8
Maharashtra	658	13.3	799	17.7
Kerala	651	13.2	779	17.3
Tamil Nadu	548	11.1	1140	25.3
**Total**	4944	100.0	4509	100.0

*ADL* Activities of Daily Living; *IADL* Instrumental Activities of Daily Living; *SC/ST* Scheduled Caste/Scheduled Tribe

About 11.4 percent of older adults were in the category of 80 + year’s age group in rural areas and urban areas the percentage was 9.3 percent. Nearly 57.8 percent of older adults in rural areas had no education in comparison to 32 percent in urban areas. About 6.1 percent of older adults in rural areas were retired in reference to 15.3 % in urban areas. Nearly, 38.1 percent of older adults in rural areas were not in a marital union in comparison to 43.2 percent in urban areas. About 22 percent of older adults from rural areas had no community involvement whereas the proportion was low in urban residents (17.7 %). Nearly, 18.6 percent and 13 percent of older adults from rural and urban areas had no trust over some someone respectively. Nearly, 23.2 percent of older adults lived alone/with the spouse in rural areas whereas the proportion was low for urban residents (19.5 %). About, 56.8 percent and 51.5 percent of older adults from rural and urban areas reported poor self-rated health status respectively. About, 7.7 percent and 59.5 percent of older adults from rural areas and 6.6 percent and 48.6 percent of older adults from urban areas reported low ADL and IADL respectively.

Table 2 represented percentage distribution of cognitive impairment among older adults by their background characteristics [rural (*n* = 4944) and urban (*n* = 4509)]. In urban areas, older adults who smoked tobacco had a higher prevalence of cognitive impairment (58.4 %). Older adults who consumed chewing tobacco had a higher prevalence of cognitive impairment (rural-68.2 % and urban-62.3 %). Alcohol consumption had a positive association with cognitive impairment among older adults from urban areas (59.6 %).

**Table 2:** Percentage distribution of cognitive impairment by background characteristics among older adults in India

**Variables**	**Rural**		**Urban**	
	**%**	***p* < 0.05**	**%**	***p* < 0.05**
**Smoking tobacco**				*
No	62.9		51.2	
Yes	62.4		58.4	
**Chewing tobacco**		*		*
No	61.2		50.1	
Yes	68.2		62.3	
**Alcohol consumption**				*
No	63.2		51.5	
Yes	58.7		59.6	
**Age (years)**		*		*
60–69	56.4		44.8	
70–79	69.7		63.2	
80+	79.1		76.5	
**Sex**		*		*
Men	55.9		44.7	
Women	69.2		58.3	
**Education**		*		*
No education	70.7		69.8	
Below 5 years	64.0		61.4	
6–10 years	42.7		37.9	
11 + years	30.7		31.8	
**Working status**		*		*
Yes	54.0		49.1	
No	68.7		58.2	
Retired	35.5		29.1	
**Marital Status**		*		*
Currently in Union	57.0		45.1	
Not in Union	72.3		61.1	
**Community involvement**		*		*
Yes	60.8		48.4	
No	70.0		68.8	
**Trust over someone**		*		*
Yes	61.2		50.6	
No	70.1		61.7	
**Living arrangement**		*		*
Others	63.9		55.3	
Living alone/with spouse	59.2		38.6	
**Self-rated health**		*		*
Good	52.7		39.6	
Poor	70.5		63.7	
**Chronic morbidity**		*		*
No	56.3		39.7	
Yes	66.3		59.4	
**ADL**		*		*
High	61.0		49.9	
Low	85.2		82.6	
**IADL**		*		*
High	51.7		45.4	
Low	70.4		59.0	
**Caste**		*		*
Non-SC/ST	60.5		49.6	
SC/ST	68.4		63.1	
**Religion**		*		*
Hindu	62.8		50.8	
Muslim	69.6		60.5	
Others	59.9		53.8	
**Wealth index**		*		*
Rich	50.7		46.2	
Middle	62.7		53.6	
Poor	68.8		64.3	
**States**		*		*
Himachal Pradesh	55.2		34.7	
Punjab	58.9		45.5	
West Bengal	83.6		78.5	
Orissa	70.0		63.5	
Maharashtra	56.7		52.4	
Kerala	69.1		61.0	
T amil Nadu	49.2		32.3	
**Total**	62.8		52.0	

*ADL* Activities of Daily Living; *IADL* Instrumental Activities of Daily Living; *SC/ST* Scheduled Caste/Scheduled Tribe; *if *p* < 0.05; %: percentage

Older adults aged 80 years and above had a higher prevalence of cognitive impairment (rural-79.1 % and urban 76.5 %). Older women had a higher prevalence of cognitive impairment (rural-69.2 % and urban-58.3 %). Older adults who had no education had the highest prevalence of cognitive impairment (rural-70.7 % and urban-69.8 %). Older adults who were reported as not working at the time of the survey had a higher prevalence of cognitive impairment (rural-68.7 % and urban-58.2 %). Being not in union (rural-72.3 % and urban-61.1 %), no community involvement (rural-70.0 % and urban-68.8 %), and no trust over someone (rural-70.1 % and urban-61.7 %) had a positive association with cognitive impairment among older adults. Older adults who reported poor self-rated health (rural-70.5 % and urban-63.7 %), chronic morbidity (rural-66.27 and urban-59.41), low ADL (rural-85.2 % and urban-82.6 %) and low IADL (rural-70.4 % and urban-59 %) had higher prevalence of cognitive impairment. Older adults from SC/ST category (rural-68.4 % and urban-63.1 %), Muslim religion (rural-69.6 % and urban-60.5 %) and poor wealth status (rural-68.8 % and urban-64.3 %) had high prevalence of cognitive impairment. Older adults from West Bengal had the highest cognitive impairment (rural-83.6 % and urban-78.5 %). Overall, older adults from rural areas had higher cognitive impairment than older adults from urban areas.

Table 3 provides logistic regression estimates for cognitive impairment among older adults in India. Model-1 was an unadjusted model which reveals that older adults who smoked tobacco had a 15 percent significantly higher likelihood to have cognitive impairment compared to older adults who did not smoke [OR: 1.15, CI:1.01–1.31]. Similarly, older adults who chew tobacco had a 62 percent significantly higher likelihood to have cognitive impairment in comparison to older adults who do not chew tobacco [OR: 1.62, CI: 1.46–1.80].

**Table 3:** Logistic regression estimates for cognitive impairment by background characteristics among older adults in India

**Variables**	**Model-1**	**Model-2**	**Model-3**	**Model-4**	**Model-5**
	**OR (95 % CI)**	**OR (95 % CI)**	**OR (95 % CI)**	**OR (95 % CI)**	**OR (95 % CI)**
**Smoking tobacco**					
No	Ref.	Ref.		Ref.	
Yes	1.15*(1.01,1.31)	1.24*(1.02,1.49)		1.22*(1.01,1.47)	
**Chewing tobacco**					
No	Ref.	Ref.	Ref.		
Yes	1.62*(1.46,1.8)	0.93(0.80,1.08)	0.92(0.81,1.08)		
**Alcohol consumption**					
No	Ref.	Ref.			Ref.
Yes	0.89(0.75,1.06)	1.30*(1.02,1.65)			1.28*(1.01–1.63)
**Age (years)**					
60–69		Ref.	Ref.	Ref.	Ref.
70–79		1.47*(1.28,1.69)	1.47*(1.29,1.69)	1.47*(1.29,1.69)	1.47*(1.29,1.69)
80+		1.97*(1.57,2.47)	1.95*(1.55,2.45)	1.95*(1.55,2.45)	1.95*(1.55,2.45)
**Sex**					
Men		Ref.	Ref.	Ref.	Ref.
Women		1.26*(1.08,1.46)	1.26*(1.08,1.46)	1.25*(1.08,1.45)	1.25*(1.08,1.45)
**Education**					
No education		3.42*(2.59,4.5)	3.41*(2.59,4.5)	3.42*(2.59,4.5)	3.42*(2.59,4.5)
Below 5 years		2.60*(1.98,3.4)	2.59*(1.97,3.39)	2.59*(1.98,3.39)	2.59*(1.98,3.39)
6–10 years		1.38*(1.08,1.77)	1.39*(1.08,1.78)	1.39*(1.08,1.78)	1.39*(1.08,1.78)
11 + years		Ref.	Ref.	Ref.	Ref.
**Working status**					
Yes		Ref.	Ref.	Ref.	Ref.
No		1.23*(1.06,1.42)	1.24*(1.07,1.43)	1.23*(1.06,1.43)	1.23*(1.06,1.43)
Retired		0.89(0.71,1.13)	0.90(0.71,1.14)	0.90(0.71,1.14)	0.90(0.71,1.14)
**Marital Status**					
Currently in Union		Ref.	Ref.	Ref.	Ref.
Not in Union		1.14*(1,1.31)	1.14(0.99,1.3)	1.14(0.99,1.3)	1.14(0.99,1.3)
**Community involvement**					
Yes		Ref.	Ref.	Ref.	Ref.
No		1.13(0.98,1.31)	1.14(0.99,1.32)	1.14(0.99,1.32)	1.14(0.99,1.32)
**Trust over someone**					
Yes		Ref.	Ref.	Ref.	Ref.
No		1.22*(1.04,1.44)	1.22*(1.03,1.43)	1.22*(1.03,1.43)	1.22*(1.03,1.43)
**Living arrangement**					
Others		Ref.	Ref.	Ref.	Ref.
Living alone/with spouse		0.94(0.81,1.09)	0.93(0.81,1.08)	0.93(0.81,1.08)	0.93(0.81,1.08)
**Self-rated health**					
Good		Ref.	Ref.	Ref.	Ref.
Poor		1.50*(1.34,1.69)	1.49*(1.32,1.67)	1.49*(1.32,1.67)	1.49*(1.32,1.67)
**Chronic morbidity**					
No		Ref.	Ref.	Ref.	Ref.
Yes		1.36*(1.20,1.54)	1.36*(1.20,1.54)	1.36*(1.20,1.54)	1.36*(1.20,1.54)
**ADL**					
High		Ref.	Ref.	Ref.	Ref.
Low		1.97*(1.49,2.59)	1.95*(1.48,2.56)	1.95*(1.48,2.56)	1.95*(1.48,2.56)
**IADL**					
High		Ref.	Ref.	Ref.	Ref.
Low		1.27*(1.12,1.44)	1.26*(1.11,1.43)	1.26*(1.11,1.43)	1.26*(1.11,1.43)
**Caste**					
Non-SC/ST		Ref.	Ref.	Ref.	Ref.
SC/ST		1.08(0.94,1.21)	1.11(0.95,1.27)	1.11(0.95,1.27)	1.11(0.95,1.27)
**Religion**					
Hindu		Ref.	Ref.	Ref.	Ref.
Muslim		0.87(0.68,1.11)	0.87(0.68,1.1)	0.87(0.68,1.11)	0.87(0.68,1.11)
Others		1.14(0.94,1.38)	1.13(0.93,1.36)	1.13(0.93,1.36)	1.13(0.93,1.36)
**Wealth index**					
Rich		Ref.	Ref.	Ref.	Ref.
Middle		1.38*(1.18,1.61)	1.38*(1.18,1.61)	1.38*(1.18,1.61)	1.38*(1.18,1.61)
Poor		1.6*(1.36,1.9)	1.58*(1.34,1.86)	1.58*(1.34,1.86)	1.58*(1.34,1.86)
**Place of residence**					
Urban		Ref.	Ref.	Ref.	Ref.
Rural		1.02(0.9,1.14)	1.02(0.9,1.15)	1.02(0.9,1.15)	1.02(0.9,1.15)
**States**					
Himachal Pradesh		Ref.	Ref.	Ref.	Ref.
Punjab		0.98(0.75,1.29)	0.98(0.75,1.29)	0.98(0.75,1.29)	0.98(0.75,1.29)
West Bengal		4.21*(3.23,5.49)	4.28*(3.28,5.59)	4.28*(3.28,5.58)	4.28*(3.28,5.58)
Orissa		1.61*(1.26,2.06)	1.63*(1.27,2.08)	1.63*(1.28,2.08)	1.63*(1.28,2.08)
Maharashtra		1.17(0.94,1.47)	1.11(0.89,1.39)	1.10(0.88,1.38)	1.10(0.88,1.38)
Kerala		2.29*(1.81,2.9)	2.35*(1.85,2.97)	2.34*(1.85,2.96)	2.34*(1.85,2.96)
Tamil Nadu		0.58*(0.46,0.73)	0.60*(0.48,0.75)	0.60*(0.48,0.75)	0.60*(0.48,0.75)
**Smoking tobacco # Alcohol consumption**					
No # No			Ref.		
No # Yes			1.39(0.93,2.07)		
Yes # No			1.24*(1.01,1.52)		
Yes # Yes			1.56*(1.21,2)		
**Chewing tobacco # Alcohol consumption**					
No # No				Ref.	
No # Yes				1.28(0.96,1.7)	
Yes # No				0.92(0.78,1.08)	
Yes # Yes				1.26(0.9,1.78)	
**Smoking tobacco # chewing tobacco**					
No # No					Ref.
No # Yes					0.90(0.77–1.08)
Yes # No					1.17(0.95–1.43)
Yes # Yes					1.15(0.82–1.60)

*ADL* Activities of Daily Living; *IADL* Instrumental Activities of Daily Living; *SC/ST* Scheduled Caste/Scheduled Tribe; *if *p*<0.05; *Ref* Reference; #: Interaction; *OR* Odds Ratio; *CI* Confidence Interval

Model-2 represents the full effect estimates. In here it was revealed that older adults who smoked tobacco had a 24 percent significantly higher likelihood to have cognitive impairment with reference to older adults who did not smoke [OR: 1.24, CI:1.02–1.49]. In this model interestingly, it was found that older adults who consumed alcohol had a 30 percent significantly higher likelihood to have cognitive impairment in comparison to older adults who did not consume alcohol [OR:1.02, 1.65]. Additionally, older adults 80 years and above [OR: 1.97, CI: 1.57–2.47], older women [OR: 1.26, CI: 1.08–1.46], respondents with no educational status [OR: 3.42, CI: 2.59–4.50] and older adults with no work [OR: 1.23, CI: 1.06–1.42] had a significant positive association with cognitive impairment. Older adults who were not in the marital union had a 14 percent significantly higher likelihood to have cognitive impairment with reference to older adults who were in marital union [OR: 1.14, CI: 1.00-1.13]. Older adults who had no trust over someone had significantly higher odds for cognitive impairment in reference to older adults who had trust over someone [OR: 1.22, CI: 1.04–1.44]. Moreover, poor self-rated health [OR: 1.50, CI: 1.34–1.69], chronic morbidity [OR: 1.36; CI: 1.20–154] low ADL [OR: 1.97, CI: 1.49–2.59] and low IADL [OR: 1.27, CI: 1.12–1.44] were significantly associated with cognitive impairment among older adults. Older adults from poor wealth status had significantly higher odds for cognitive impairment than older adults from rich wealth status [OR: 1.6CI: 1.36, 1.9].

Model-3, model-4, and model-5 were the models with interaction effects. It was revealed that older adults who had a habit of smoking tobacco and consuming alcohol simultaneously were at brisk of worst cognitive outcomes i.e. those older adults had 56 percent significantly higher likelihood to have cognitive impairment than older adults who neither smoke tobacco nor drink alcohol [OR: 1.56, CI: 1.21-2.00]. Similarly, the results were for older adults who chew tobacco and consume alcohol simultaneously however the results were not significant [OR: 1.26, CI: 0.9–1.78]. Additionally, older adults who had a habit to smoke and chew tobacco had a 15 % higher likelihood to have cognitive impairment than older adults who neither smoke nor chew tobacco [OR: 1.15; CI: 0.82–1.60].

## Discussion

The increased life expectancy led populations to use substances for longer until the compounding effect of substance and declining health leads to significant morbidity and mortality [55–57]. Similarly, smoking and chewing tobacco increase morbidity and result in bad health outcomes both among adults and older individuals [58, 59]. In agreement with this, the present study also confirms the first hypothesis we tested that alcohol consumption and tobacco use are positively associated with cognitive impairment in later years.

The natural aging process brings notable cognitive challenges. And the cognitive impairment adversely affects wellbeing and is a strong predictor for chronic disease progression, and subsequent mortality [60]. On the other hand, while substance use generally declines in later adulthood, even small amounts of alcohol use can have serious consequences [61]. Our results suggest that risk factors for cognitive impairment among older adults include higher age and female sex, while a higher educational level, working status, community involvement are protective factors. Compared to the results from studies conducted in the urban population in India [41, 62, 63], the rates of cognitive impairment in rural areas is substantially higher, considering that a rural location is an established risk factor for dementia in Asian countries [64–66]. Also, the regression results show that lower educational attainment has been linked to lower cognitive ability. More years of education translates into a greater cognitive reserve [63, 67]. Older men and women who are not currently in the marital union were found to have lower cognitive ability compared to those who are currently in the marital union. It is consistent with earlier studies suggesting that older adults in any stressful marital relationship or widowhood may be as likely to engage in health-related substance use behaviour’s due to increased stress from loss of financial and/or emotional resources and face more severe cognitive impairments [66, 68].

Similar to the patterns of cognitive impairment observed in Western studies that found an association of cognitive functioning with chronic diseases and somatic comorbidities [69, 70], our results also found that cognitive impairment was significantly associated with chronic morbidity. In concordance with previous studies in India [41], our study also observed that a substantial proportion of older adults with higher functional ability (ADL and IADL) reported lower cognitive impairment supporting the concept of successful cognitive aging. A couple of cohort studies also showed a positive association between self-rated health and current cognitive function among older adults and found poor self-rated health as a predictor of higher cognitive impairment in the advanced ages [71, 72]. Furthermore, a recent study also found that older adults with chronic kidney disease and poor functional and general health status were at increased risk for cognitive impairment [73]. Consistently, the results of the current study showed that those who reported poor self-rated health had lower cognitive ability than their counterparts.

Our findings also support the notion that smoking or chewing tobacco seems to be a risk factor for cognitive impairment. Current analysis found that smokers were more likely to be cognitively impaired than never smokers after adjusting for control variables such as age, sex, education, working status, and other social and health-related variables. Further, this study also observed alcohol use is positively associated with cognitive impairment. This is consistent with the view that alcohol consumption and substance use, which contribute to vascular diseases [19], could increase the risk for dementia and cognitive impairment [74, 75]. On the other hand, it is also likely that those individuals who maintain cognitive function may display more adaptive qualities and abstain from substance use. Besides, the potential confounding effects of depressive symptoms, prescriptive drugs, and other substances are not taken into consideration in the current analysis. And finally, we cannot say for certain that maintaining cognitive function is causally related to not smoking or not consuming alcohol.

In the final model with interaction analyses, a significant interaction effect between the use of smoked tobacco and alcohol consumption on cognitive impairment was found. The older adults who were smokers and consumed alcohol exhibited greater cognitive deficits than those only smokers or who only consumed alcohol. Surprisingly, the odds of cognitive impairment were higher among those who used smokeless tobacco and consumed alcohol than those who consumed alcohol only unlike those who used smokeless tobacco only. Hence, though the results were not significant, the interaction effect also showed that smokeless tobacco is a predictor of cognitive impairment if the person consumed alcohol and used smokeless tobacco or used any form of tobacco that is, smoked and smokeless tobacco jointly leading to the partial confirmation of our second hypothesis. However, different mechanisms may underlie the adverse effects of heavy drinking and the beneficial effects of light to moderate drinking, and such mechanisms may also partly explain why certain differences are reported in decline in cognitive ability in later years.

The study suffers from certain limitations that need to be mentioned. Firstly, the results cannot be generalized for present scenario as the survey was conducted in 2011. Secondly, seven states of India that represent different regions of India were accounted to provide the estimates at national level. Therefore, one should be cautious while generalizing it for pan India. Another limitation of the study is that since it was cross-sectional rather than longitudinal, the findings should be interpreted with caution and it cannot attribute a final direction to the relationship of substance use and cognitive impairment. Moreover, given the benefits of moderate alcohol consumption and the risks of excessive drinking and smoking, the interplay between alcohol use and other substance use and cognitive ability requires further study to formulate a more crystallized understanding of the effect of substance use on cognitive health outcomes [16, 19, 76]. Finally, the dose and duration and consumption patterns of alcohol and tobacco have not been taken into account in the current study.

However, apart from these limitations, the BKPAI survey is one of the limited surveys in the context of India which provides such reliable estimates at the national level. Moreover, it is one of the most recent surveys in the Indian scenario which provide estimates on substance use among older adults and also report their cognitive functioning ability.

## Conclusions

The findings of this study have implications for societies that are aging and consuming alcohol or chewing/smoking tobacco. When planning geriatric health care for older adults, priority must be given to older-older, women, illiterate, and those older adults who are socially less involved and have poor health outcomes, as they are more vulnerable to impaired cognitive function. Thus, the encouragement of older people to stop using smoked and smokeless tobacco could be considered as part of a strategy to reduce the incidence of cognitive impairment.

Moreover, despite the benefit of earlier diagnosis is established [56], most of the older adults with cognitive impairment do not receive a diagnosis and if they do, it happens late in their disease course. Hence, appropriate measures should be taken for the detection of early stages of cognitive decline in older individuals and efforts should be made to improve the availability and quality of care for dementing older adults. Furthermore, the preventive measures for cognitive impairment should focus on countering the risk factors suggested by the current evidence such as alcohol drinking, smoking and chewing tobacco, and other factors too. And other measures also include increased taxation of tobacco products and bans on its advertisements. Meanwhile, more research is warranted to identify the modifiable risk factors for declining cognitive ability among older adults.

## Data Availability

The study utilizes a secondary data which is available only on request from director@isec.ac.in or india.office@unfpa.org.

## Abbreviations

OR: Odds Ratio
CI: Confidence Interval
ADL: Activities of Daily Living
IADL: Instrumental Activities of Daily Living
SC: Scheduled Caste
ST: Scheduled Tribe
